# A False-Positive HIV Test: Severe Lupus Flare in Disguise

**DOI:** 10.7759/cureus.24349

**Published:** 2022-04-21

**Authors:** Eric Lam, Najia Sayedy, Javed Iqbal

**Affiliations:** 1 Internal Medicine, Nassau University Medical Center, East Meadow, USA; 2 Pulmonary and Critical Care, Nassau University Medical Center, East Meadow, USA

**Keywords:** false-positive, intensive care, hiv, immunocompromised, systemic lupus erythematosus

## Abstract

Systemic lupus erythematosus (SLE) and human immunodeficiency virus (HIV) infection have significant overlapping clinical features, making diagnosis challenging. We report a case of new-onset SLE initially mistreated as HIV infection due to a false-positive fourth-generation HIV antigen/antibody (Ag/Ab) test.

A young female in her 30s presented with fatigue, oral thrush, and a positive HIV Ag/Ab combo test. She was started on fluconazole and highly active antiretroviral therapy (HAART), but deteriorated with recurrent fevers and worsening mental status, requiring ICU admission. Surprisingly, her HIV confirmatory tests were negative, but rheumatologic serologies were positive. The overall clinical, laboratory and biopsy results confirmed the diagnosis of SLE. She was treated with pulse steroid therapy and immunosuppressive agents with marked improvement and was subsequently discharged.

Rarely do SLE patients present with false-positive HIV tests, thus masking and delaying treatment for critical SLE. Clinicians should understand the limitations of screening tests and have high suspicions and consider the diagnoses of both diseases.

## Introduction

Systemic lupus erythematosus (SLE) and human immunodeficiency virus (HIV) infection are both systemic disorders that may affect all organs in the body. Both diseases present with nonspecific, constitutional symptoms including fever, fatigue, weight loss, myalgia, and arthralgias, making the differentiation between the two diseases challenging. Both SLE and HIV-infected patients may develop severe disease with multiorgan dysfunction requiring ICU admission. Rarely, SLE patients may even present with false-positive HIV screening tests due to the presence of autoantibodies [1]. Due to the difference in nature of the two disorders, it is essential to make an accurate diagnosis to initiate the appropriate treatment. Prior reports described false positivity in third-generation HIV screening tests in SLE patients [1]; however, to our knowledge, there is no literature reporting false-positive HIV diagnosis with the highly sensitive and specific fourth-generation HIV antigen/antibody (Ag/Ab) combo test in SLE patients. We present a case of new-onset SLE that was initially mistreated as HIV infection due to a false-positive fourth-generation HIV Ag/Ab combo test.

This article was previously presented as a meeting abstract at the CHEST 2021 Annual Meeting, virtually on October 18, 2021.

## Case presentation

A young female in her 30s presented to the hospital with worsening generalized fatigue for three weeks. She reported diffuse muscle aches, bilateral ankles, wrists joint swelling, and oral thrush with associated dysphagia for ten days. She denied any history of intravenous (IV) drug use or previously HIV test. The patient stated she was only sexually active with her husband. On admission, she was afebrile and hemodynamically stable. Pertinent physical examination findings included oral thrust and white tonsillar exudates. Decreased breath sounds were noted on auscultation of the lower lung fields, associated with pleuritic pain. Tenderness was elicited upon palpation of bilateral wrists and ankles joints, which was associated with joint swelling and stiffness. Hands and toes were warm to touch without ulcer or joint deformity. No rash was appreciated on skin examination.

**Figure 1:**
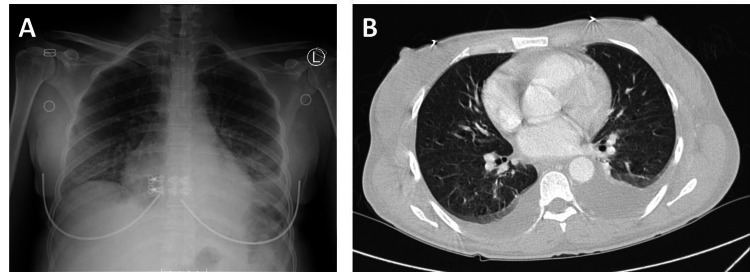
Radiographic studies of the patient (A) Chest x-ray shows hazy opacity at the lung bases compatible with effusions. (B) CT thorax demonstrates bilateral pleural effusions, left greater than right, with adjacent passive atelectasis in the lower lobes.

Initial laboratory testing showed leukopenia and lymphopenia (white blood cell count 3.37 K/mm^3^ and absolute lymphocyte count 0.93 K/mm^3^). The preliminary HIV Ag/Ab combo test (Atellica IM CHIV, Siemens Healthineers, Erlangen, Germany) was positive. She was also found to have a decreased absolute CD4 count of 247 cells/mcl (normal range: 490-1740 cells/mcl). Chest imaging revealed bilateral pleural effusions (Figure 1).

Additional findings revealed nephrotic-range proteinuria. She was started on fluconazole for the oral thrush and highly active antiretroviral therapy (HAART) for HIV infection. 

Her hospital course was complicated by recurrent high fever episodes and a gradual decline in mental status. Despite empiric broad-spectrum antibiotics and HAART, she continued to deteriorate with worsening mental status; she also developed hematemesis. She became hemodynamically unstable with a fever of 104 degrees Fahrenheit, significant tachycardia of 150s beats per minute, and tachypnea of 40 breathes per minute, requiring endotracheal intubation and mechanical ventilation for acute respiratory failure. Surprisingly, the confirmatory HIV-1 antigen and HIV-1/HIV-2 antibodies returned negative. Given her clinical presentation, meeting at least 4 of 11 of the American College of Rheumatology (ACR) criteria for SLE [2], including arthritis, evidence of pleural effusions with pleuritic pain, nephrotic-range proteinuria, and leukopenia in the setting of negative HIV confirmatory test, a rheumatologic workup was prompted. She was found to have high antinuclear antibodies (ANA) 1:1280 with speckled pattern, low complements C3 and C4, positive anti-Smith antibodies, ds-DNA antibodies, ribonucleoprotein (RNP), anti-Sjogren's syndrome A (SSA) and anti-Sjogren's syndrome B (SSB), rheumatoid factor (RF), and positive anticardiolipin IgM, IgG, and IgA. All clinical and laboratory findings suggested the diagnosis of SLE (Table 1).

**Table 1:** Pertinent laboratory tests of the patient HIV=human immunodeficiency virus, Ag/Ab = antigen/antibody, Qt RT-PCR= quantitative real-time-polymerase chain reaction, abs= absolute, ESR= erythrocyte sedimentation rate, CRP= c-reactive protein, ANA= antinuclear antibodies, RNP=ribonucleoprotein, SS= Sjögren's-syndrome, Ig= immunoglobulin, CCP= cyclic citrullinated peptide, B2= beta-2.

Laboratory test	Result	Unit	Normal Range
Infectious Disease			
HIV Ag/Ab Combo	Preliminary: Reactive		Negative
HIV RT-PCR and sequencing	Not detected		
HIV viral Qt RT-PCR	RNA not detected		
Cell count and differentials			
Abs Neutrophil	2.26	K/mm3	[1.80-7.00 K/mm3]
Abs Lymphocyte	0.93	K/mm3	[1.5-4.00 K/mm3]
CD 3+ Abs	468	cells/mcL	840-3060 cells/mcL
CD 4+ Abs	247	cells/mcL	[490-1740 cells/mcL]
CD 8+ Abs	218	cells/mcL	[180-1170 cells/mcL]
Chemistry			
ESR	36	mm/Hr	[0-20 mm/Hr]
CRP	3.3	mg/dL	[0.0-0.9 mg/dL]
Complement C3c	18	mg/dL	[83-193 mg/dL]
Complement C4c	4	mg/dL	[15-57 mg/dL]
Rheumatoid Factor	15	IU/mL	<14 IU/mL
Urine protein-creatinine ratio	6.43	mg/mg	
Immunology			
ANA titer	1:1280	titer	<1:40
ANA pattern	Nuclear, Homogeneous		
DNA ab (ds) Crithidia titer	1:1280	titer	<1:10
RNP Extract Nuclear Ab	>8.0	AI	<1.0
Smith Ab	>8.0	AI	<1.0
SS-A	>8.0	AI	<1.0
SS-B	>8.0	AI	<1.0
CCP IgG	16	Units	<20 units
Cardiolipin IgM	>150	MPL	<=12 MPL
Cardiolipin IgG	62	GPL	<=14 GPL
Cardiolipin IgA	>150	APL	<=11 APL
B2 Glycoprotein I IgM	25	SMU	<=20 SMU
B2 Glycoprotein I IgG	<9	SGU	<=20 SGU
Lupus Anticoagulant	Negative		Negative
Direct Coombs	Positive		Negative

**Figure 2:**
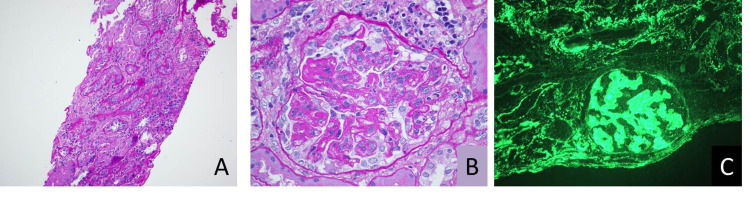
Renal biopsy images Renal biopsy pathologic studies of the patient are most consistent with a diffuse endocapillary proliferative, segmental membranous, and focal sclerosing immune complex-mediated glomerulonephritis, favoring the diagnosis of diffuse proliferative and membranous lupus nephritis (class IV and V). (A) Tubular atrophy and interstitial fibrosis. (B) Endocapillary proliferative glomerulonephritis. (C) Immunofluorescence microscopy staining for IgG showing glomerular and extra-glomerular deposits.

Bronchoscopy with bronchoalveolar lavage (BAL) was performed to rule out diffuse alveolar hemorrhage in the setting of recent hemoptysis and positive lupus serology; bronchoscopy was unremarkable for any hemorrhage, but BAL revealed pneumocystis jirovecii (PCP), with negative viral cultures. Esophagogastroduodenoscopy showed no thrush beyond the oral cavity and revealed mild gastric erosion suggestive of gastritis. To confirm the initial false-positive preliminary HIV test findings, HIV viral load real-time PCR (Taqman, Roche Diagnostics) and genotype analysis were sent; both returned negative. Renal biopsy was performed and immunofluorescence microscopy showed granular global mesangial and glomerular capillary wall deposits which stained strongly positive for IgG, IgM, IgA, C1q, kappa and lambda, and mildly positive for C3, which is consistent with the full-house pattern of lupus nephritis. Further, the findings of diffuse endocapillary proliferative, segmental membranous, and focal sclerosing immune complex-mediated glomerulonephritis favored the diagnosis of class IV and class V lupus nephritis (Figure 2). 

The patient was started on IV pulse methylprednisolone therapy with 1 g daily for three days and trimethoprim/sulfamethoxazole for PCP prophylaxis, and HAART was promptly discontinued. The patient’s symptoms drastically improved after pulse methylprednisolone treatment. In less than 24-hours of initiation of treatment, her hemodynamics, mental and respiratory status significantly improved and she was successfully extubated. She was transitioned to IV methylprednisolone 30 mg every eight hours and was transferred to the medical ward for the management of SLE and nephritis. She was transitioned to oral prednisone 60 mg daily, initiated on mycophenolate mofetil 1 g orally two times a day and hydroxychloroquine sulfate 200 mg orally daily, and was subsequently discharged with outpatient follow-up.

## Discussion

SLE and HIV infection have significant overlapping clinical features such as presentations of fever, oral lesions, arthralgias, and malaise, with laboratory results including leukopenia, lymphopenia, and the production of autoantibodies. Initial screening HIV tests may even be falsely positive due to antiphospholipid autoantibodies, which makes it difficult to differentiate the two diseases. A lack of response to initial treatment in our patient prompted the consideration of an alternative diagnosis. Clinicians should understand the overlapping presentations of SLE and HIV infection and confirm the diagnosis with appropriate laboratory and diagnostic tests.

SLE is a systemic disease that may involve multiple organs with a spectrum of disease presentation ranging from mild to severe. While arthritis and arthralgias are the most common presentation, clinicians must keep in mind that mucocutaneous, cardiac, pulmonary, renal, and hematologic manifestations are also frequently reported [2]. The classic mucocutaneous presentation is cutaneous lupus erythema or the "butterfly rash”; however, mucous membrane lesions are also common. Cardiac manifestations include pericardial effusion, pericarditis, endocarditis, and valvular disorders. Pulmonary manifestations of SLE include pleural effusions, interstitial lung disease, and alveolar hemorrhage. Renal involvement in SLE is also common, with the presentations of proteinuria, hematuria, and the risk of development of rapidly progressive glomerulonephritis and renal failure. Hematologic abnormalities in SLE may affect all three cell lines, causing leukopenia, anemia, and thrombocytopenia. Patients with SLE and multiple organs involvement may develop severe disease presentation requiring ICU admission. The most common causes of ICU admission in SLE patients are infection, renal dysfunction, cardiovascular disease, and coagulopathies [3].

The diagnosis of SLE is confirmed by meeting the 2019 European League Against Rheumatism (EULAR)/American College of Rheumatology (ACR) classification criteria. These criteria provide a point system for diagnosing SLE and was designed to improve sensitivity for early diagnosis of new-onset of SLE [2]. To make the diagnosis, the patient must meet the entrance criteria of a positive ANA titer of > 1:80, meeting at least one clinical criterion, and having a score of 10 or more (total score of 51) in the seven clinical domains (constitutional, hematologic, neuropsychiatric, mucocutaneous, serosal, musculoskeletal, and renal) and three immunology domains (antiphospholipid antibodies, complement proteins, and SLE-specific antibodies) [2]. It is important to note that the presence of antiphospholipid antibodies (anti-cardiolipin antibodies, anti-beta-2GP1 antibodies, and lupus anticoagulant) is one of the criteria in the immunology domain above. In fact, studies have reported that antiphospholipid antibodies were detected in up to 47% of SLE patients [4]. As a result, it is important to screen all SLE-suspected patients for the presence of antiphospholipid antibodies, as this finding significantly increases the risks of thromboembolic diseases [5].

Antibody screening tests, such as HIV antibody screening tests, may return false positive due to autoantibodies and cross-reactivity in patients with SLE. Case reports described false-positive HIV screening test results in the setting of SLE with third-generation HIV screening tests [1]. The fourth-generation HIV Ag/Ab combo test is highly sensitive and specific (both >99%) in patients with chronic HIV infection as it detects both HIV p24 antigen and antibodies to HIV-1 and HIV-2. A preliminary positive HIV Ag/Ab combo test may be due to the detection of antigen, antibodies, or both, and will require further confirmatory testing with HIV-1/HIV-2 differentiation assay - as in our patient’s positive preliminary positive test but negative confirmatory test. HIV RNA real-time-PCR testing is the most sensitive assay and may also be used to confirm the diagnosis. A negative confirmatory HIV-1/HIV-2 differentiation assay and negative HIV RNA real-time-PCR result confirmed the initial positive test result was a false positive. However, due to the low false-positive rate, a positive HIV screening test should prompt the initiation of HAART. Additionally, HAART should be continued until the confirmation of a false-positive screening test, as immune reconstitution inflammatory syndrome is an important differential diagnosis that may present within one week after initiation of HAART. While SLE patients may present with a false-positive HIV test, patients with active HIV infection can present with autoantibodies such as ANA and anticardiolipin antibodies [6]. Additionally, cases of coexistence of SLE and HIV infection are very rare but have been reported [7]. As a result, clinicians should have a high suspicion of either or both diseases based on the overall clinical presentation, and must not rely solely on the results of screening tests.

Critically ill SLE patients with major organ involvement are treated with high-dose IV methylprednisolone (up to 1 g/day) to decrease systemic inflammation. Other immunosuppressive agents such as cyclophosphamide and mycophenolate may also be used but should be considered with caution due to their cytotoxicity and side-effect profile. The detection of antiphospholipid antibodies is associated with an increased risk of thrombotic events. Low-dose aspirin may be used in these patients for the prevention of thrombosis. In asymptomatic patients, the role of anticoagulation is unclear and not recommended. Anticoagulation should only be initiated if a thromboembolic event is confirmed. In cases of severe antiphospholipid syndrome with systemic thrombosis and end-organ damage, plasma exchange and intravenous immunoglobulin (IVIG) are indicated. In SLE patients presenting with cough, dyspnea, hemoptysis, with low or decreasing hemoglobin and unilateral or bilateral diffuse opacities on chest radiograph, diffuse alveolar hemorrhage should be considered. Bronchoscopy with sequential BAL must be performed promptly to confirm the diagnosis due to the acute disease progression. In addition to hemodynamic support, aggressive treatment with a combination of pulse steroid therapy and immunosuppressive agents, such as cyclophosphamide, rituximab, mycophenolate mofetil, and plasmapheresis may improve outcomes [8].

## Conclusions

We highlight a case of new-onset SLE initially mistreated as HIV infection. SLE and HIV infection have significant overlapping clinical features and laboratory findings. Rarely, SLE patients may present with a false-positive HIV screening test, thus masking and delaying treatment for systemic SLE. Clinicians should understand the limitations of initial screening tests and have high suspicion and consider the diagnoses of both SLE and HIV infection.
